# Development of the *Astyanax mexicanus* circadian clock and non-visual light responses

**DOI:** 10.1016/j.ydbio.2018.06.008

**Published:** 2018-09-15

**Authors:** Inga A. Frøland Steindal, Andrew D. Beale, Yoshiyuki Yamamoto, David Whitmore

**Affiliations:** a Department of Cell and Developmental Biology, University College London, 21 University Street, London WC1E 6DE, UK; b MRC Laboratory of Molecular Biology, Francis Crick Avenue, Cambridge, CB2 0QH, UK

**Keywords:** *Astyanax mexicanus*, Circadian clock, Development, DNA repair, Non-visual light detection

## Abstract

Most animals and plants live on the planet exposed to periods of rhythmic light and dark. As such, they have evolved endogenous circadian clocks to regulate their physiology rhythmically, and non-visual light detection mechanisms to set the clock to the environmental light-dark cycle. In the case of fish, circadian pacemakers are not only present in the majority of tissues and cells, but these tissues are themselves directly light-sensitive, expressing a wide range of opsin photopigments. This broad non-visual light sensitivity exists to set the clock, but also impacts a wide range of fundamental cell biological processes, such as DNA repair regulation. In this context, *Astyanax mexicanus* is a very intriguing model system with which to explore non-visual light detection and circadian clock function. Previous work has shown that surface fish possess the same directly light entrainable circadian clocks, described above. The same is true for cave strains of *Astyanax* in the laboratory, though no daily rhythms have been observed under natural dark conditions in Mexico. There are, however, clear alterations in the cave strain light response and changes to the circadian clock, with a difference in phase of peak gene expression and a reduction in amplitude. In this study, we expand these early observations by exploring the development of non-visual light sensitivity and clock function between surface and cave populations. When does the circadian pacemaker begin to oscillate during development, and are there differences between the various strains? Is the difference in acute light sensitivity, seen in adults, apparent from the earliest stages of development? Our results show that both cave and surface populations must experience daily light exposure to establish a larval gene expression rhythm. These oscillations begin early, around the third day of development in all strains, but gene expression rhythms show a significantly higher amplitude in surface fish larvae. In addition, the light induction of clock genes is developmentally delayed in cave populations. Zebrafish embryonic light sensitivity has been shown to be critical not only for clock entrainment, but also for transcriptional activation of DNA repair processes. Similar downstream transcriptional responses to light also occur in *Astyanax*. Interestingly, the establishment of the adult timing profile of clock gene expression takes several days to become apparent. This fact may provide mechanistic insight into the key differences between the cave and surface fish clock mechanisms.

## Highlights

• Non-visual light sensitivity is developmentally delayed in cave strains of *Astyanax.*
• Neither strains of Astyanax show clock gene oscillations in dark raised larvae.
• The timing of the circadian molecular clock rhythm is delayed by 6-h in Pachon.
• Expression *CPD photolyase* is raised in constant darkness in Pachon.
• The clock has gained greater regulation over light-responsive genes in cave strains.

## 1 Introduction

Most animals and plants live on a rhythmic planet, with regular and predictable periods of light and dark. As a result, they possess an endogenous circadian clock that synchronizes their physiology and behaviour with the environmental light-dark cycle. Light is the most significant signal for setting the clock, and animals possess a variety of non-visual light detection mechanisms to achieve this. Most of what we know about teleost clocks and light-sensitive biology comes from studies in zebrafish (Tamai et al., 2005., Tamai et al., 2007; Vallone et al., 2004; Weger et al., 2011; Whitmore et al., 1998; Whitmore et al., 2000). All zebrafish tissues are directly light-sensitive and contain a circadian pacemaker, which means that all tissues can detect light and set the circadian clock without the need for eyes or a centralized neural clock (Whitmore et al., 2000). Clocks contained within specific tissues control the rhythmic physiology of those tissues, though the requirement for whole body coordination of these daily oscillators in fish is not yet clear. As a consequence of this direct cellular light senstitivity, the tissues and cells of the fish body must contain the relevant photopigments or opsins, and the intracellular signalling pathways necessary to set this clock. This is, in fact, the case with fish expressing up to thirty-two non-visual opsins (Davies et al., 2003). Different tissues express various combinations of these opsins, the functional consequence of which is not yet completely clear, but it certainly provides fish tissues with a remarkable potential to absorb and respond to light of various intensities and wavelengths (Davies et al., 2011, Davies et al., 2003).

With such a remarkable whole body, light sensitivity, it is not surprising that setting the clock is not the only function of environmental light detection. It is clear that zebrafish light sensitivity activates numerous cell signalling events, which impact a variety of fundamental cell processes, including cell cycle regulation through the clock, metabolic processes and cell communication, but perhaps the most strongly light-regulated events are those relating to DNA repair (Dekens et al., 2003, Dickmeis et al., 2007, Hirayama et al., 2009, Tamai et al., 2004, Tamai et al., 2005, Tamai et al., 2012). Not only is light necessary for the protein function of DNA repair enzymes, such as the photolyases, but it is also for their transcriptional activation. If a fish is not exposed to light, then it is unable to turn on a wide range of pathways essential for DNA repair. It is clear, therefore, that light responsiveness and the presence of a clock are fundamental aspects of fish physiology.

In this context, the study of non-visual light detection and clock biology is extremely intriguing in species such as *Astyanax mexicanus* (Beale et al., 2016). Over the past few million years, groups of *Astyanax mexicanus*, has been isolated from neighbouring rivers in underground caves in the North East of Mexico (Gross, 2012). As a result, we can today find over 30 distinct populations of *A. mexicanus* in numerous isolated caves. All of these populations have evolved and adapted to a life in complete darkness. Adaptations to the dark include the loss of eyes and pigment, as well as changes in metabolic rates, activity and the loss of sleep activity/circadian rhythms to varying degrees (Beale et al., 2013, Jeffery, 2009, Gross et al., 2009, Jaggard et al., 2018, Protas et al., 2006, Protas et al., 2007, Yoshizawa et al., 2015). What makes the *A. mexicanus* such an excellent model for studying not only adaptive and regressive evolution, but also adaptations of light and clock biology to a dark environment, is that the founding species of river fish are still found in abundance in the rivers of Mexico. The surface fish and the cave strains of *A. mexicanus* have not fully speciated, and can therefore be crossed in the laboratory to produce F1 hybrids. It is therefore possible to determine molecular adaptations to the constant darkness, by directly comparing the founding river fish with the isolated cave populations (Bradic et al., 2012, Dowling et al., 2002, Strecker et al., 2003, Strecker et al., 2004).

One would expect the fundamental aspects of light and clock biology to be very similar between surface strains and those described in zebrafish, if only because both live and have evolved in a rhythmic light-dark river environment. However, cave strains offer a much more interesting scenario, where the existence and role of light and clock biology is obviously far from clear, considering the long period of evolution in a completely dark environment. Several previous studies have addressed this issue to some extent in adult animals, though not to date during embryo development. From an activity perspective, cave strains of *Astyanax* lack any robust day-night rhythms in activity that are seen in surface populations, being both effectively continuously active and not showing signs of classical sleep behaviour (Duboue et al., 2011). At the molecular clock level, cave strains in the laboratory are still capable of showing rhythmic, daily oscillations in gene expression (Beale et al., 2013). However, these clock rhythms show certain, specific alterations between surface and cave strains. Cave populations possess molecular clock rhythms with lower amplitude than surface fish, and the phase or daily timing of these rhythms is clearly delayed by up to 6 h. However, in the caves themselves, in North eastern Mexico, to date there is no evidence of any molecular clock rhythms, and in fact the expression levels of several clock components appears to be repressed. Under natural conditions, there is no evidence to date that they employ a rhythmic molecular clock to control timed aspects of their physiology.

Though there are clear mutations in the circadian clock mechanism in cave strains, perhaps the largest changes are seen in the response of these animals to light (Beale et al., 2013). In cave strains, light-inducible genes that are essential for clock entrainment are already highly transcribed in the dark. Cave strains look “molecularly” as if they are living under constant light conditions when in fact living in constant darkness. Consequently, the degree of apparent light activation is greatly reduced. As these genes, such as the light-inducible *period* genes and *cryptochrome 1a* (*cry1a*), are transcriptional repressors, one hypothesis is that their basally raised expression levels are in part the reason for the reduced amplitude of the cave strain clock, as well as the delayed phase seen in the molecular mechanism. This basal activation of light responsive genes is not only restricted to clock genes, but genes that encode the light responsive DNA repair genes, photolyases, also show increased levels of expression in the dark. As DNA repair is a highly light-dependent process in fish, this change in the regulation of these genes to being expressed at high levels in the dark in cave strains is probably a very critical adaptation for these animals to survive in the cave environment.

The above changes in clock and light biology have been explored in adult *Astyanax mexicanus*, but never during the early stages of embryo development. Yet in zebrafish, it has been shown that both the clock and light have a major impact on the process of embryo development, and the regulatory genes involved in embryogenesis (Dekens and Whitmore, 2008, Laranjeiro and Whitmore, 2014). The molecular clock appears to begin to oscillate early in zebrafish development with the first peak in *period1* gene expression seen at 27 h post fertilization. Acute non-visual light sensitivity can be detected even earlier by between 6 and 9 h post fertilization and before the differentiation of any classical light responsive structures in the embryo (Tamai et al., 2004, Dekens and Whitmore, 2008). Photolyases involved in DNA repair become transcriptionally activated at this developmental stage also, and a lack of light exposure during embryo development leads to a dramatic increase in larval mortality when these dark raised embryos are exposed to environmentally stressing conditions, such as UV light exposure. The clock controls the expression of many genes known to be important in the process of embryo development, including the regulation of genes critical in the regulation and timing of the cell cycle, such as *p20/p21* (Laranjeiro et al., 2013). Interestingly, the rhythmic regulation of these downstream/output genes often does not occur until day 3–4 of development, and raises the possibility that a fully functional circadian clock system is not present until these later stages of embryo development.

Considering the relevance of non-visual light detection and circadian rhythmicity to development in zebrafish, the obvious question arises about how these processes function during the development of *Astyanax mexicanus* comparing both surface and cave strains. What are the embryonic differences in early light sensitivity between strains? Does a molecular clock become established as early as detected in zebrafish, and is there a difference between surface and cave populations? Do cave strains develop a circadian clock in the same manner as surface fish, and are the differences reported in adult *Asytanax* present immediately in cave strain embryo development? Furthermore, how does this impact the critical regulation of DNA repair activation during development? In this study, we will address each of these issues in *Astyanax mexicanus*, exploring the differences between surface and cave strains. We demonstrate that surface fish are acutely light responsive from the earliest stages of development, but that this light sensitivity appears to be delayed in cave strains. This difference is not dependent upon alterations in pineal physiology, as this light response occurs globally in most cells in *Asytanax mexicanus*, as previously described for zebrafish. A very shallow circadian oscillation can be detected in surface embryos during the first two days of development, with no rhythm present in cave strains, but in both cases a more robust circadian clock begins to function on the third day of development. Interestingly, the balance of light *versus* circadian clock regulation appears to differ for classically light-regulated genes, such that the clock impacts these rhythms more strongly in cave strains than surface fish. As a result, one can detect more robust daily rhythms in the *period2* genes and *CPD photolyase* in cave populations than surface embryos on a dark-light cycle. This may initially seem rather unexpected, but may reflect an evolutionary switch from light to clock gene regulation of critical genes in a constant dark environment.

## 2 Materials and methods

### 2.1 Biological materials and embryo maintenance

Adult surface, Pachón and Chica cavefish maintained at 22–25 °C were exposed to a 14:10-h photoperiod (light intensity 300–500 µW/cm^2^). The fish were mated every 3 weeks by *in vitro* fertilization (IVF) and 12–20 embryos were collected in E3 fish water (5 mM NaCl, 0.17 mM KCl, 0.33 mM CaCl2, 0.33 mM MgSO4, 0.00001% Methylene Blue) in 25 ml flasks. The embryos were kept at 25 °C in a thermostatically controlled water bath and placed on a 12:12 DL cycle or in constant darkness within 1–2 h of fertilization. 12–20 embryos were harvested at 6-h intervals in TRIzol Reagent (Invitrogen), homogenized and stored at − 20 °C. Total RNA was extracted following the manufacturer's instructions. For acute light pulse experiments, embryos were kept in constant darkness and given a 3-h light pulse at 5hpf, 14 hpf and 23 hpf or kept in the dark as a control. Embryos were then collected, homogenized and total RNA extracted as above. All animals were maintained in a Home Office approved facility and handled in accordance with the Animal Welfare Act of 2006.

### 2.2 Real-Time Quantitative PCR (RT-qPCR)

cDNA was synthesised from 2 μg of RNA using Superscript II Reverse Transcriptase (Invitrogen) with random hexamers and oligo dT primers. RT-qPCR was performed on a C1000 Touch™ Thermal Cycler with the CFX96™ Optical Reaction Module (Bio-Rad) using KAPA SYBR FAST qPCR mix (Kapa Biosystems) in technical triplicates with gene specific-primers (see Supplementary information) at a concentration of 500 nM. ΔCt was determined using *rpl13α* or *EF1α* as a reference gene and relative expression levels between surface and cavefish were compared directly using the ΔΔCt method

### 2.3 Whole mount *in situ* hybridisation

At the assigned timepoints, embryos were fixed in 4% PFA/PBS overnight at 4 °C. Early embryos were dechoreonated. Embryos were washed 4 times with PBS before storage in 100% MeOH at − 20 °C. All further steps were conducted at room temperature unless otherwise stated. Embryos were rehydrated in a series of washes with 75% MeOH in PBT (PBS + 0.01% Tween-20), 50% MeOH in PBT, and 25% MeOH in PBT and twice in PBT. Embryos were then treated with 10 µg/ml proteinase K for 5 min, washed with PBT twice, before fixation with 4% PFA/PBS for 20 min. After five PBT washes, embryos were washed with HYB+ solution and incubated in HYB+ for at least 2 h at 65 °C. Digoxigenin-labelled (DIG)-labelled probes (antisense and sense) were synthesised from 1 µg of linearised plasmid DNA containing a 559 bp fragment of *per2b* using T7 or SP6 polymerase (Promega) and digoxigenin-labelled dUTP (Roche). DIG-labelled probes were prepared by denaturing in HYB+ ( 5xSSC, 0.1% Tween-20, 5 mg/ml torula (yeast) RNA, 50 μg/ml heparin) at 80 °C for 2 min before being diluted to 1 µg/ml in HYB+ . Embryos were incubated with DIG-labelled RNA sense or antisense probe in HYB+ overnight at 65 °C with gentle shaking.

After hybridisation, the embryos were successively washed at 65 °C with HYB+ , 50% HYB+ /2 × SSC, 2 × SSC, and twice in 0.2 × SSC before being cooled to room temperature, and subject to a further three washes with PBS. After washing, embryos were incubated with 2% Blocking Agent (Roche) in maleic acid buffer (MAB) for at least 3 h. The block was replaced with anti-DIG-alkaline phosphatase (1:5000) in 2% Blocking Agent in MAB, and the embryos were incubated overnight at 4 °C.

The embryos were subsequently washed four times in PBS, equilibrated in BM staining buffer, and incubated with BM purple in the dark at room temperature until the colour was sufficiently developed. Finally, embryos were washed twice with PBT and refixed with 4% PFA/PBS overnight at 4 °C.

The *In situ* hybridisation signal was quantified by densitometry in ImageJ (1.50i, Schneider et al. (2012). Images were converted to 8-bit grayscale and a region of interest, encompassing the embryo, was drawn using the Specify tool. Optical density was calculated using the “Analyze tool” from the peak of the profile plot of each sample after enclosing the peak and eliminating background noise.

### 2.4 Statistical analysis

The data in this study are presented as the mean± s.e.m. (*n* > 3) and were analysed using a Student's *t-*test or analysis of variance (http://www.physics.csbsju.edu/stats). *P* < 0.05 was considered significant.

## 3 Results

### 3.1 Light responsiveness in early embryos

The non-visual light response develops very early in small teleosts, well before the differentiation of the retina or pineal gland, and light exposure within the first few days of development has been shown to be crucial for embryonic development, survival and fitness in many species (Gavriouchkina et al., 2010, Martín-Robles et al., 2012, Tamai et al., 2004, Weger et al., 2011). What adaptations and changes in the light input pathway are found in cavefish embryos to compensate for developing in a dark environment?

Light regulates and sets the circadian clock through the transcriptional activation of light sensitive genes, which themselves are typically transcriptional repressors (Carr and Whitmore, 2005, Hirayama et al., 2005, Pando et al., 2001, Tamai et al., 2007, Vallone et al., 2004, Ziv et al., 2005). Work in zebrafish has shown that the *per2* and *cry1a* are involved in the entrainment of the clock to light in the embryo and adult fish, which makes these three light-inducible genes excellent markers for the onset of light-sensitivity (Dekens and Whitmore, 2008, Tamai et al., 2007, Ziv and Gothilf, 2006). The coding regions of the core clock and light-inducible genes are highly conserved between the different populations of *Astyanax mexicanus*, which allows the use of the same gene-specific qPCR primers for all populations of fish.

**Fig. 1:**
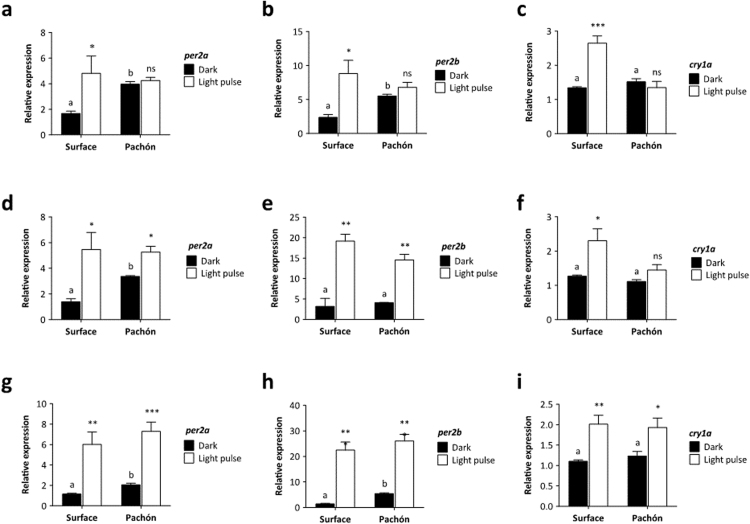
Acute light induction of clock genes is slower to develop in Pachón cavefish than surface fish. Surface and Pachón embryos were maintained in constant darkness until a 3-h light pulse was given at different developmental stages. Expression of *per2a*, *per2b* and *cry1a* was determined by qPCR in light-pulsed and dark control samples and normalised to the reference gene *rpl13α*. Relative expression was calculated using the ΔΔCt method. (a-c) Light pulse given at 5 hpf, (d-f) light pulse given at 14 hpf, (g-i) light pulse given at 23 hpf. Dark and light-induced levels were compared using a Student's *t*-test (unpaired, two tailed; *, p < 0.05; **, p < 0.01; ***, p < 0.001; significant differences at p < 0.05 in dark samples indicated by different lower case letters), Data represent the mean± SEM for between 3 and 5 embryo samples.

To determine when cavefish become light responsive, Surface and Pachón embryos were raised in complete darkness and given a 3 h light-pulse at 3 different times during the first day of development (5 hpf, 14 hpf and 23 hpf). Light sensitivity is established very early in surface fish. Both *per* genes, as well as *cry1a* are induced by light as early as 5–8 hpf (Fig. 1a-c). At 14–17 hpf and 23–26 hpf, we see further increases in light-inducible transcription of *per2a* and *per2b* mRNA transcript. In comparison, the Pachón embryo is much slower in developing a light response. There are no significant increases in any of the light sensitive genes in 5–8 h old Pachón embryos (Fig. 1a,b,c). At 14–17 hpf, we see a robust light response in both the *per2* genes in Pachón embryos, but yet we do not see a *cry1a* response to light until the very end of Day 1 (Fig. 1. i). Interestingly, throughout development, the basal levels (expression in DD) of the *per2* genes are raised in Pachón cavefish compared to surface fish (Fig. 1), except *per2b* 14–17 hpf (Fig. 1. e) where there is a non-significant expression difference between DD samples in Pachón and surface embryos. Presumably, it is these raised levels in the dark that prevent any additional measureable light-induction. We also observe this increased basal transcription of the *per2b* genes in adult Pachón fish (Beale et al., 2013). However, there is an even stronger *per2b* fold basal induction in Pachón embryos compared to adults. The basal levels of *cry1a* however, are the similar for cave and surface strains (Fig. 1f,i).

**Fig. 2:**
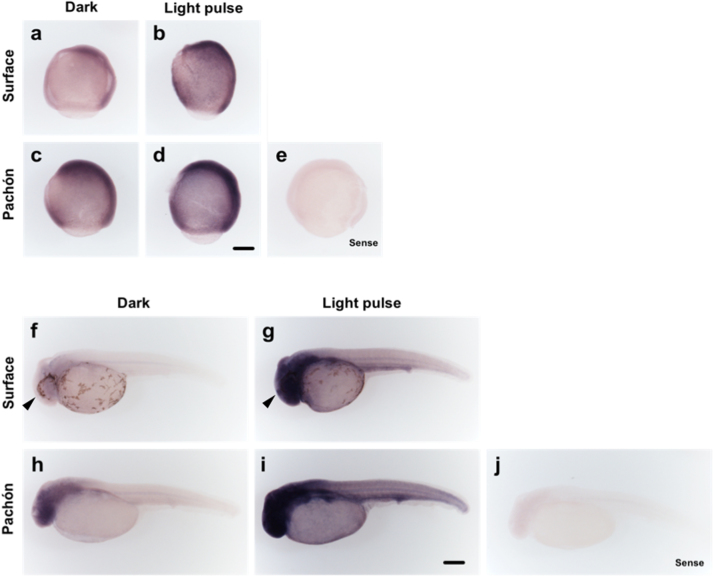
Acute light induction develops within the first day of development in Pachón cavefish. Surface and Pachón embryos were kept in constant darkness until a 3-h light pulse was given beginning at (a-d) 5 hpf and (f-i) 23 hpf. Expression of *per2b* mRNA was analysed by *in situ* hybridisation in light-pulsed and dark control samples, with the same detection time for all treatments. (e and j) *per2b* sense control for embryos at 8 hpf and 26 hpf respectively. Black arrow indicates the position of the pineal gland. Scale bar, 0.4 mm.

Although it is clear that a light response exists in early Pachón embryos (from both the differences between *per1* expression rhythms in LD and DD (Fig. 4) and significant acute responses to light at 14–17 hpf) (Fig. 1. d,e), a light response similar to that seen in Surface fish is only present in Pachón embryos at 23–26 hpf (Fig. 1g-i). This maturation of the acute light response (when *per2a*, *per2b* and *cry1a* are all significantly induced) coincides with the development of a functional pineal gland in *Astyanax* (Yoshizawa and Jeffery, 2008). In addition, *per2* mRNA expression is rapidly induced in response to light in zebrafish, with significant changes detected in the pineal gland (Vatine et al., 2009, Ziv et al., 2005). It could, therefore, be argued that much of this embryonic light response is pineal dependent. Therefore, we analysed the expression of *per2b* mRNA using whole mount *in situ* hybridisation to examine whether the high induction gained at 23 hpf in Pachón embryos is due to pineal-enhanced expression. *In situ* hybridisation confirmed the increased expression of *per2b* after light exposure at 5 hpf and 23 hpf in surface embryos (Fig. 2a and b, and f and g). This expression difference is only present in Pachón embryos when the light pulse is given at 23 hpf (Fig. 2h and i), similar to the results obtained by qPCR. *Per2b* is clearly expressed at raised levels in Pachón embryos compared to surface fish in the dark controls, as seen by qPCR, and which is apparent in these samples at both time points (Fig. 2c and h). At 26 hpf, the expression in both surface and Pachón embryos is ubiquitous throughout the embryo, though clearly somewhat stronger in the head region of the larvae. The ubiquitous expression of *per2b* at 26 hpf observed by *in situ* hybridisation, and the clear light response present before 17 hpf, show that it is not the pineal gland alone that mediates the development of the light-induction of clock genes in Pachón. The mechanism of light detection is present throughout the embryo and is not restricted to central photoreceptive structures, in both surface and cave strains of *Astyanax*.

### 3.2 Cave-cave hybrid fish do not show a rescued light-response in epiboly-stage embryos

Cave populations of *Astyanax mexicanus* have arisen at least five times independently (Bradic et al., 2012) and show remarkable convergence in characteristics, such as eye loss and pigmentation. Furthermore, a unique and valuable feature of *Astyanax* is that cavefish from different caves can still be crossed to examine cave phenotypes by complementation tests. It is clear that development of the light response in Pachón embryos is delayed compared to surface fish. Can this delay in light responsiveness be rescued in a F1 generation created by mating two cave strains?

**Fig. 3:**
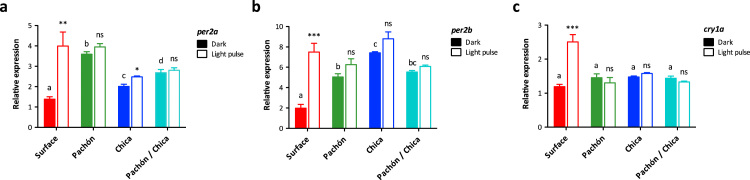
Acute light-induction at 5 hpf is not rescued in a cave-cave hybrid. Surface, Pachón, Chica and Pachón/Chica hybrid embryos were kept in constant darkness until a 3-h light pulse was given at 5 hpf. Expression of (a) *per2a*, (b) *per2b* and (c) *cry1a* was determined by qPCR in light-pulsed and dark control samples and normalised to the reference gene *ef1α*. Dark and light-induced levels were compared using a Student's *t*-test (unpaired, two tailed; *, p < 0.05; **, p < 0.01; ***, p < 0.001) and dark levels were compared by ANOVA and Newman-Keuls multiple comparison test (significant differences at p < 0.05 in dark samples indicated by different lower case letters). Data represent the mean± SEM for 3–5 embryo samples.

In order to test whether early light sensitivity can be rescued by another cave strain, we examined the induction by light of multiple clock genes in Pachón, Chica and Pachón-Chica hybrid embryos by raising the embryos in the dark and giving a 3-h light-pulse at 5 hpf. *Per2a* show a very small (1.23 fold), but significant induction in Chica embryos, yet there is no induction of *per2b* or *cry1a* (Fig. 3). Furthermore, Chica embryos also show high expression of both *per* genes in DD, similar to that described for Pachón (Fig. 3a, b). Interestingly, we do not see any rescue of light responses in the “cave-cave” hybrid. The small *per2a* increase seen in Chica is no longer present, yet we still see an increased amount of *per2* transcript in the “cave-cave” hybrid animals (Fig. 3a, b).

### 3.3 Clock entrainment

The circadian clock of zebrafish begins on the first day of development (Dekens and Whitmore, 2008, Ziv and Gothilf, 2006). The core circadian clock mechanism is generated by a transcriptional-translational negative feedback-loop, which is highly conserved in all vertebrates (Harmer et al., 2001, Takahashi, 2004, Wilsbacher and Takahashi, 1998). *Per1* is one of the key genes of the core clock, and shows high-amplitude circadian oscillations in entrained adult *Astyanax mexicanus*, making *per1* an excellent marker of clock function (Tamai et al., 2007, Park et al., 2007, Velarde et al., 2009; Martín-Robles et al., 2012; Beale et al., 2013).

**Fig. 4:**
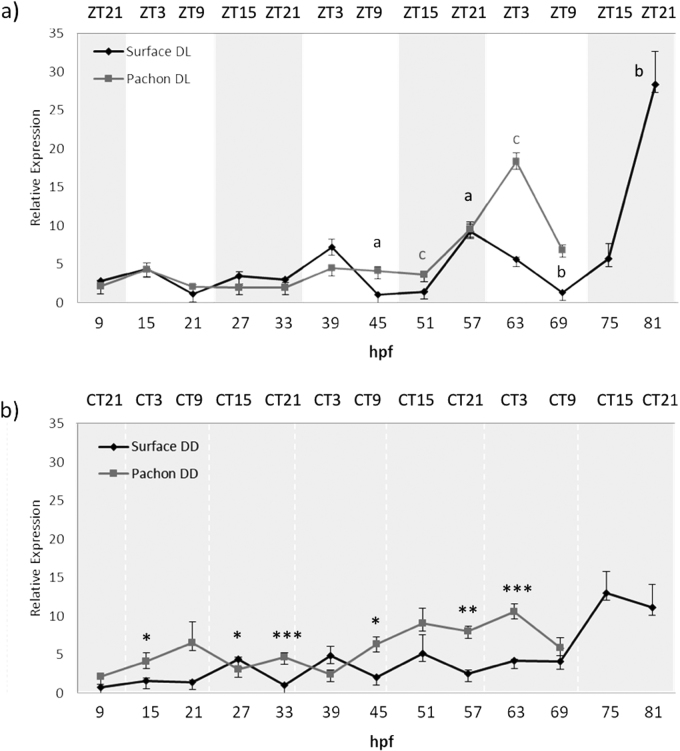
*Per1* is rhythmically expressed during development. Embryos were sacrificed every 6 h for 3–4 days from 9 h post fertilization (hpf). *Per1* mRNA levels were measured by qPCR, normalised to the reference gene *RLP 13α* and relative expression was calculated using the ΔΔCt method. a) Surface and Pachón were entrained to a 12:12 DL cycle. Grey and white shaded areas indicate dark and light periods respectively. Zeitgeber time (ZT) on the upper x-axis gives hours after the light has come on. P < 0.05 significance between peak and trough is indicated by lower case letters in black for Surface and dark grey for Pachón. b) Surface and Pachón raised in the dark. Upper x-axis denotes circadian time (CT) for the dark samples. Levels of *per1* mRNA under constant dark were compared at each timepoint using a Student's *t*-test (unpaired, two tailed; *P < 0.05;<0.01; ***P < 0.001). Data represent the mean ± SEM for 3 biological replicates.

To determine when the *Astyanax* circadian clock starts, Surface and Pachón embryos were entrained to a 12–12 h dark-light (DL) cycle and embryos were harvested at 6-h intervals for the first 3–4 days of development. It is worth noting that we employed a reverse light-dark cycle for these experiments, compared to most studies in the literature, to match with the natural spawning times of the cave populations, which occur primarily during the night. Samples were analysed by RT-qPCR to determine the levels of *per1* mRNA. During the first two days of development, surface fish *per1* show a low amplitude rhythm that peaks 3 h after the onset of light (ZT3) (Fig. 4a). However, from the third day of development, we start to observe a higher amplitude rhythm, were the peak has now shifted to ZT21. There is no apparent *per1* rhythm in surface embryos raised in constant dark (Fig. 4b). In comparison, we see an almost flat expression of *per1* for the first two days in Pachón embryos, and no robust *per1* rhythm until is observed until 51 hpf, with a peak at ZT3 (Fig. 4a). There is also no apparent *per1* rhythm in Pachón embryos maintained in DD (Fig. 4b).

There are clear and consistent differences between the rhythms seen in surface and Pachón embryo populations, which we also see in adult cavefish. *Per1* peak expression occurs 6 h later in Pachón compared to surface embryos, which means the timing and entrained phase of the *per1* rhythm is the same in adult and embryo for both strains.

### 3.4 Alterations in rhythmic expression of *per2a* and *per2b* transcriptional repressors in developing cave strains

We have previously hypothesised that the differences in rhythm amplitude and the phase angle of *per1* expression between cave and surface strains is likely to be due to changes within the core clock mechanism that generates the oscillation, as well as alterations in the light input pathway. So, what changes do we see in the expression of the *per2* light-induced transcriptional repressors in the developing embryo?

**Fig. 5:**
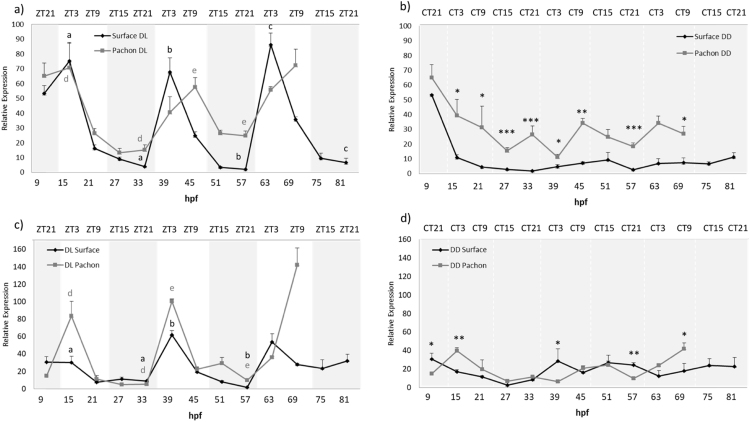
Light inducible genes show altered expression patterns in Pachón embryos. Surface and Pachón embryos were sacrificed every 6 h for 3–4 days from 9 h post fertilization (hpf). *Per2a* and *per2b* mRNA levels were measured by qPCR, normalised to the reference gene *RLP 13α* and relative expression was calculated using the ΔΔCt method. a, b) *Per2a* expression in surface and Pachón embryos raised on a 12:12 DL cycle and DD, respectively. c, d) *Per2b* expression in surface and Pachón embryos raised on a 12:12 DL cycle and DD, respectively. P < 0.05 significance between peak and trough on a DL cycle is indicated by lower case letters in black for Surface and dark grey for Pachón. Expression of both *per2* genes was compared between surface and Pachón cavefish at each time point by using a Student's *t*-test (unpaired, two tailed; *P < 0.05;<0.01; ***P < 0.001). Data represent the mean± SEM for 3 biological replicates.

Both cave and surface strains show highly rhythmic and light induced expression of *per2a* from the first day of development (Fig. 5a). *Per2a* transcripts peak at ZT3 with an average 21.9-fold difference in expression between peak and trough expression in surface fish. In comparison, Pachón *Per2a* peaks 6 h later, at ZT9 and only shows an average 3.5-fold change between peak and trough values (Fig. 5a). This dramatic difference in day-night levels is once again due to raised basal levels of *per2a* transcripts in the cave population. It is also interesting to note that in DD, the amount of *per2a* transcript is on average 2.9 times as abundant in Pachón as in surface embryos (Fig. 5b). We also note that there are similar high amounts of maternally deposited RNA for *per2a* in both populations of cavefish.

*Per2b* shows similar rhythmic expression to *per2a* in surface fish, however, we do not see a marked increase in *per2b* transcript during the first light phase in these animals (Fig. 5c). In contrast, Pachón show a higher amplitude of *per2b* expression at ZT3 (15 hpf and 39 hpf), but at 69 hpf, *per2b* expression shifts 6 h and peaks at ZT9. In complete darkness, the expression of *per2b* is flat, but not raised in the cave strain (Fig. 5d). Furthermore, it is also interesting to note that there are barely any maternal deposits of *per2b* mRNA transcript in both strains, compared to *per2a.*

### 3.5 DNA repair gene expression is altered in cave strains

DNA repair, using enzymes such as CPD photolyase (*CPD phr*), is one of several important processes that are light induced in small teleosts (Tamai et al., 2004, Gavriouchkina et al., 2010, Weger et al., 2011). Are the changes in light sensitivity that we describe for the clock also impacting the regulation of DNA repair gene transcription in the developing embryo?

**Fig. 6:**
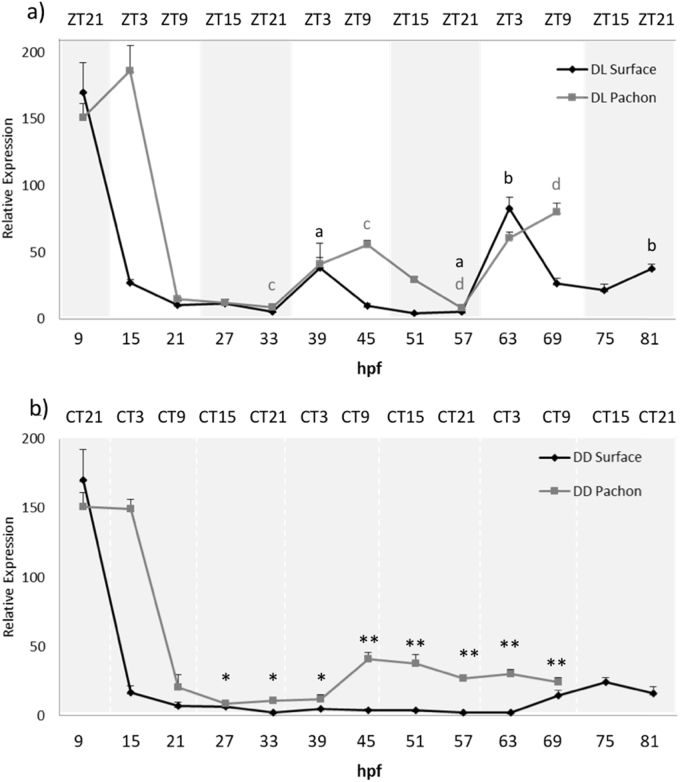
Expression of the DNA repair enzyme CPD is differentially expressed in Pachón embryos raised both on a DL cycle and in DD. Surface and Pachón embryos were sacrificed every 6 h for 3–4 days from 9 h post fertilization (hpf). *CPD* mRNA levels were measured by qPCR, normalised to the reference gene RLP 13*α* and relative expression was calculated using the ΔΔCt method. a) *CPD phr* expression in embryos raised on a 12:12 DL cycle. P < 0.05 significance between peak and through is indicated by lower case letters in black for Surface and dark grey for Pachón. b) *CPD phr* expression in embryos raised in constant darkness. Expression levels of *CPD photolyase* were compared between surface and Pachón in DD at each time point by using a Student's *t*-test (unpaired, two tailed; *P < 0.05; **P < 0.001). Data represent the mean± SEM for 3 biological replicates.

Entrained surface embryos show high amplitude rhythms of *CPD phr* expression with peaks at ZT3 from day 2, with very little transcript present at ZT9, ZT15 and ZT21 (Fig. 6a). In Pachón, the *CPD phr* peaks 6 h later at ZT9. Interestingly, there is also a high amount of *CPD* transcript expressed at ZT3, which means there is much more CPD transcript present at any time in the Pachón embryo, compared to the surface embryo (Fig. 6a). It is also clear that in the dark, there is a higher basal expression of *CPD phr* in Pachón embryos, compared to surface (Fig. 6b). In order to explore the maternal deposition levels of *CPD* transcript, and other clock genes, we examined the amounts of mRNA present in unfertilized oocytes for Pachón and surface strains (Supplementary Fig. 1). There is a considerable amount of *CPD phr* deposited maternally in both strains. This remains high in both strains even after the maternal RNA for the *period* genes has dropped, at the time that would typically correspond to the transcriptional activation of the zygotic genome. Interestingly, in both DL and DD, there is a raised level of *CPD phr* in 15 h old Pachón embryos, while in surface embryos the amount is considerably lower (Fig. 6). This might suggest that either the maternal *CPD* is more stable in the cave strains and remains present for longer, or that the zygotic transcriptional activation of *CPD* is more sustained in the early cave populations. Interestingly, there is considerably higher amounts of maternal RNA for the *period* genes in Pachón than surface fish. The biological significance of this is far from clear and will require further examination.

## 4 Discussion

### 4.1 Advent of light induction

*Astyanax mexicanus* has established itself not only as a major model system with which to study evolution, but also to examine circadian clock function and light regulated biology (Beale et al., 2013, Bradic et al., 2012, Strecker et al., 2003). How the circadian clock develops or “begins to tick” is a fundamental question in circadian biology. Clock function and light dependent biology is absolutely crucial for healthy development in most teleost species, so what happens in an animal that develops in darkness and in fact never experiences light? Are there differences in the development of the clock mechanism in cave strains, which might shed light on how the circadian system has altered following evolution in a dark environment?

In this study, we have explored clock and light responsive biology during the early developmental stages of *Astyanax mexicanus.* We have shown that there are differences in the development of the light response of surface and cavefish, as well as differences between the amplitude and of phase of light-inducible genes under rhythmic conditions. Surface fish become light responsive during the first 5–8 h of development (Fig. 1a-c), similar to that reported for zebrafish, and before the development of any tissue or light responsive organs, such as eyes or the pineal gland. In contrast, the fold induction of light-inducible genes is reduced in Pachón embryos and is slower to develop. The *per2* genes do not show a light induction until 14–17 h (Fig. 1d, e). In the case of the *per2* genes, this lack of induction is most likely the consequence of the fact that basal expression levels are raised in early Pachón embryos, leaving little range for a further light-driven increase. One argument is that this indicates that there may not be a delay in the development of actual light sensitivity. However, the data collected for *cry1a* expression does not suffer from the same issues, with basal levels being very similar between surface and Pachón, yet there is a clear developmental delay with *cry1a* induction not showing any light response until 23–26 hpf (Fig. 1i). As such, it is interesting to note that light induction in Pachón develops at different stages for *per2* genes and *cry1a*, and that the increase of light induced transcript appears later for *cry1a* than *per2*. In addition, the absolute fold induction for both *per2* genes increases with developmental age, possibly reflecting a maturation of the light signalling process, whereas *cry1a* induction stays constant at around 2-fold in surface embryos (Fig. 1). These results strongly suggest that there is a different mechanism involved in *per2* and *cry1a*'s response to light in Pachón. This might not be unexpected as the transcriptional regulation of *per2* and *cry1a* in zebrafish has also previously been shown to differ (Mracek et al., 2012).

The underlying mechanism for this delayed development in light sensitivity is not yet clear, but could reflect alterations in any aspects of the signalling pathway, including the expression of the relevant opsins. What is clear is that *per2b* expression, when examined by *in situ* hybridisation, shows that there is a global response to light in both in surface and Pachón larvae (Fig. 2). At 26 hpf, the expression in both surface and Pachón embryos is ubiquitous throughout the embryo, with only a slight increase in staining in the pineal gland. We also observe a clear light response in embryos present before 17 hpf, which shows that it is not the pineal gland nor a delay in its development that mediates the light-induction of clock genes in *Astyanax* (Fig. 2f-i). This is in line with and expected from observations in zebrafish, where the mechanism of light detection is present throughout the embryo and is not restricted to central photoreceptive structures. This does not imply that the light responsiveness of the pineal gland is irrelevant, just that light sensitivity is a global fish-tissue phenomenon.

### 4.2 Absence of ‘rescue’

Cave-cave hybrids are able to ‘rescue’ a number of degenerate features of cave animals, such as Pachón/Tinaja and Tinaja/Molino, which produce embryos with larger eyes than either parent, and Molino/Curva hybrids which are extensively pigmented (Borowsky, 2008, Jeffery, 2009). On the contrary, crosses of Pachón/Molino and Pachón/Japonés cavefish are albino, like their parents (Protas et al., 2006). These complementation tests reveal that, in addition to the independent evolutionary origin, eye regression in Pachón, Tinaja and Molino is predicted to be due to separate genetic mechanisms. Conversely, the genetic basis for albinism in Pachón, Molino and Japonés is the same: a mutated form of *oca2* (Protas et al., 2006).

Using a similar experimental paradigm, we examined the genetic basis of the absence of light-response in early embryos. Hybrid F1 embryos of Pachón and Chica are not light-responsive between 5 hpf and 8 hpf, just like the Pachón and Chica F0 embryos themselves (Fig. 3). Whilst we have not been able to identify the nature of the mechanism that is responsible for this phenotype yet, it does suggest that Pachón and Chica have alterations in the same gene or pathway. This is a remarkable result as Pachón and Chica cavefish are predicted to have separate evolutionary origins, and have been geographically isolated from each other for several million years, and so this result means a similar alteration in the light input pathway has evolved convergently, in the same way as albinism (Bradic et al., 2012, Protas et al., 2006). In the future, it would be interesting to expand this number of crosses between many different cave populations, firstly to determine if the surface-like response is recovered, and secondly to see if the alterations of the light input pathway in the different cavefish strains are due to selection or drift.

### 4.3 Per2 gene rhythmic expression

Both the *per2* genes show different, but altered expression patterns during Pachón development, which may indicate that these two similar genes act in different ways in the clock mechanism. The *per2a* expression pattern is very different in Pachón compared to surface fish, both in the entrained state and in a constant dark environment (Fig. 5a, b). The basal levels of *per2a* expression are clearly raised in constant darkness in Pachón, as well as on a DL cycle. (Fig. 4a, b). On a light-dark cycle, surface fish *per2a* shows a strong acute light-driven rhythm, which is absent in the dark. This is in contrast to the entrained rhythm in Pachón, which shows a “smoother” expression pattern, rather than an acute and immediate response to light seen in surface fish, and with a clear 6-h delay in the timing of peak levels (Fig. 5a, b). Effectively, this means that there is more *per2a* transcript produced in the embryo on average during a day for Pachón. In the case of *per2b* expression, the daily rhythm in expression appears, if anything, to be more robust in Pachón than surface fish larvae, though this will require further statistical confirmation. A 6-h change in phase between the strains is not detected until day 3 of development, one day later than for *per2a* (Fig. 5c, d).

What is the possible explanation of this rather complicated looking set of expression data? There is clearly a difference between the expression patterns between *per2a* and *per2b*, which is especially apparent in constant darkness. *Per2a* shows significantly raised levels of transcript whilst *per2b* basal expression is the same as surface (Fig. 5). Taken together with the difference of expression patterns in DL, this clearly shows the two genes are regulated differently in Pachón. This might indicate that the two different isoforms of *per2* are likely to play different roles in the clock and light-input pathway, although the precise mechanism is not clear. It seems apparent that both *per2* genes in surface fish are primarily light-regulated. This is shown by the low expression in constant dark, and the nature/waveform of the rhythm on a light-dark cycle. However, in adult Pachón, the light inducible genes *per2b* and *CPD* continues to oscillate in the dark after being exposed to a LD – cycles (experiments of *per2a* were not performed at the time) (Beale et al., 2013). Furthermore, the phase difference between surface and cave strain suggests that the *per2* genes are now much more under the control of the circadian clock itself, in addition to an acute light input. This hypothesis also fits with the development of the clear 6-h phase difference, seen on day 2 or 3 of development, when a robust clock is beginning to function in *Astyanax*. At first this would seem to make little sense, as exposing cave strains to a light-dark cycle is obviously an anomalous situation, and one they would not normally experience in nature. However, in cave populations clearly light-regulated genes would never normally be induced, due to the total darkness. So, by evolving additional regulation by clock-components, even in the dark, expression of these genes will be turned on, though presumably they will not show daily rhythms in the cave. These cave strains are using clock regulatory factors not to generate a rhythm, but to increase tonically the expression of genes that would normally be turned on by the light. The phase differences we report are, in fact, effectively an artefact of “seeing” a rhythmic light-dark cycle. The proof of this, of course, would be to find a change in the promoter/regulatory regions of the *per2* genes that gives them this circadian “gain of function”.

### 4.4 Development of the clock

In zebrafish, the molecular clock becomes functional from the first day of development (Dekens and Whitmore, 2008). In cavefish, however, we do not observe a robust daily oscillation in *per1* gene expression in either of the strains until the third day of development. The surface strains show shallow *per1* oscillations from the first day of development that peaks at ZT3. However, during day three, we see a change in phase angle and amplitude similar to what we see in adults. This change in clock phase during development to an adult timing condition is quite an unexpected and unusual observation. Though we do not yet know the precise mechanism underlying this phase shift, it suggests that the clock mechanism itself undergoes a developmental maturation over the first three days of development, with perhaps not all of the components to form a robust clock being present until day three onwards. Though the zebrafish clock appears to develop earlier than the *Astyanax* pacemaker, there is also evidence that it too might not become fully functional until day 3–4 of development, as many clock output genes do not become rhythmic until this considerably later developmental stage (Laranjeiro and Whitmore, 2014). It is, however, clear that light has an impact on *per1* expression during the first day of development, as we can observe an increase in *per1* transcript in entrained embryos as opposed to those in constant dark. Pachón do not show any prominent *per1* peaks during the first two days of development, although light must have some impact, as *per1* expression in DD is different and much “noisier”. It is not until day 3 that we see oscillations starting at 63 hpf, peaking at ZT3, the same phase as in adult Pachón.

In the wild, the expression of *per1* in adult cavefish is very low. This is likely due to the increased expression of CRY1a and PER2 proteins that act as strong repressors of CLOCK-BMAL transcriptional activity, which in turn dampen *per1* gene expression rhythms, reducing amplitude and slowing circadian oscillations. In contrast to adult cavefish, Pachón embryos show noisy expression of *per1*, looking as if the clock is running asynchronously. This correlates with *per2b* also showing “noisy/erratic” expression in DD. Previous work in zebrafish has shown that constant light ‘stops’ the circadian oscillator. *Per2* and *cry1a* are involved in the entrainment of the clock to light and the maintenance of high amplitude rhythms, while overexpression of both these genes mimics constant light conditions (Dekens and Whitmore, 2008, Tamai et al., 2007, Ziv and Gothilf, 2006). Therefore, the reduced amplitude and timing of the embryonic cavefish clock could, like adults, be a consequence of changes within the light input pathway. Analysis of the coding sequence has shown a high conservation between the different cave strains (Beale et al., 2013). Therefore, again we hypothesize that the changes in *per2* transcript that we see in Pachón are likely to be due to changes in the promoter regions, enhancing the expression of per2a in the absence of light.

Our working hypothesis is that evolution has acted to convert the regulation of purely light responsive genes in surface fish over to effectively clock-regulated genes in cave strains. Even though these animals do not live in a rhythmic environment this adaptation enables the positive transcriptional regulation of clock elements to tonically increase the gene expression of genes that would otherwise be “switched off” in the transition from life in the river to life in the cave. This is certainly reflected in expression of the typically light-inducible *per* genes, but what about other teleost light-regulated processes, such as DNA repair. From our results, examining CPD photolyase expression, it is clear that the expression profile in Pachón closely resembles that of *per2a*. In the case of *CPD phr* there is also a large amount of maternally deposited transcript in both strains, which actually remains raised in Pachón for six hours longer. In surface fish the transcription of this gene is solely light-dependent, and so levels are very low under constant dark conditions. In Pachón the timing of the *CPD phr* rhythm is delayed by 6 h, and the expression levels on a light-dark cycle are consequently significantly greater. Again, this supports the idea of the circadian clock now playing a significant role in regulating the expression of this gene. The result of this, as discussed above, mean that under natural constant dark conditions the levels of *CPD phr* are raised. This fact is undoubtedly very important during embryo development in the darkness of the cave, as if this evolutionary change had not occurred then the ability of these animals to repair damaged DNA would be greatly impaired. These results fit with our previous data collected in adult *Astyanax mexicanus*, and show that this important biological adaptation occurs during the earliest stages of embryo development. A future full transcriptomic analysis of cave strains, both rhythmically and in response to light, will demonstrate the full extent of these evolutionary changes. As a full comparison of genomic sequences will allow us to demonstrate that there are clear alterations in the enhancer/regulatory regions of these central clock and light esponsive genes.
