# Supplementary material for: Development of the Astyanax mexicanus circadian clock and non-visual light responses
Source: Dev Biol. 2018 Sep 15;441(2):345–54. doi: 10.1016/j.ydbio.2018.06.008 (PMC6141809; doi:10.1016/j.ydbio.2018.06.008)
Supplement: Supplementary file 1 — Supplementary material [file mmc1.docx]

**6. Supplementary**

**Supplementary 1 Deposits of maternal mRNA in Surface and Pachón embryos**
We compared gene expression between Surface and Pachón unfertilized oocytes (0 hpf) to 9 hpf old embryos kept in constant darkness. Expression of *per1,* *per2a*, *per2b* and *CPD* was determined by qPCR and normalised to the reference gene *rpl13α*. Relative expression (RE) was calculated using the ΔΔCt method. (a-d) Comparison of gene expression between maternally deposited mRNA at 0 hpf and mRNA at 9hpf in Surface strain in black and Pachón strain in light grey. Significance between 0 hpf and 9 hpf old embryos in the strain as well as significance between Surface and Pachón 0 hpf, were compared using a Student’s t-test (unpaired, two tailed)
 *, p<0.05; **, p<0.01; ***, p<0.001. Data represent the mean ± SEM for between 3 oocyte/embryo samples.
